# Public Knowledge and Perceptions about the Emerging Human Mpox in Jordan: A Cross-Sectional Study

**DOI:** 10.3390/tropicalmed8010041

**Published:** 2023-01-05

**Authors:** Rana K. Abu-Farha, Karem H. Alzoubi, Tareq L. Mukattash, Rama Alkhawaldeh, Muna Barakat, Samar Thiab

**Affiliations:** 1 Department of Clinical Pharmacy and Therapeutics, Faculty of Pharmacy, Applied Science Private University, Amman 11931, Jordan; 2 Department of Pharmacy Practice and Pharmacotherapeutics, University of Sharjah, Sharjah 27272, United Arab Emirates; 3 Department of Clinical Pharmacy, Faculty of Pharmacy, Jordan University of Science and Technology, Irbid 22110, Jordan; 4 Department of Pharmaceutical Chemistry and Pharmacognosy, Faculty of Pharmacy, Applied Science Private University, Amman 11931, Jordan

**Keywords:** mpox, human mpox virus, knowledge, perception, outbreak, Jordan

## Abstract

Hundreds of human mpox cases are spreading outside of Western and Central Africa, which could be considered a significant world health problem. In this study, we sought to assess public knowledge and perceptions of human mpox. The study was a cross-sectional survey conducted in Jordan in September–October 2022. All participants were approached through social media portals. A total of 1195 participants agreed to participate in this study. The participants showed a low median knowledge score about human mpox (4.0 out of 15.0, IQR = 5.0), with only 27.9% of the participants (n = 334) correctly identifying that human mpox is not a bacterial disease, and around half of them (n = 589, 49.3%) knowing that this disease affects both genders. Higher monthly income (≥400 JD/month) was significantly associated with better knowledge about the disease. Social media was the most comprehensive source of information about mpox (n = 1018, 85.2%). Finally, 57.3% of the participants (n = 685) believed that mpox would affect social and economic life, like the COVID-19 pandemic. These findings highlight the urgent need for systematic strategies that should be promoted to increase public knowledge of mpox, which will be critical in improving the capacity to respond to the disease.

## 1. Introduction

Human mpox is a re-emerging zoonotic infectious disease. The disease is caused by a virus classified within the genus orthopoxvirus of the Poxviridae family and results in smallpox-like symptoms in humans [1]. The mpox virus was initially identified in 1958 at a Danish laboratory for monkeys in Copenhagen, Denmark [2]. However, it was first confirmed as a human disease in 1970, when a nine-month-old child was suspected of having a smallpox infection at a hospital in the Democratic Republic of Congo (formerly known as Zaire) [2].

Human mpox virus can spread from animal to human by close contact with infected live or dead animals [3]. Furthermore, the human mpox virus can be transmitted from human to human through direct contact with respiratory secretions, skin lesions, and objects that have been contaminated with the infection [3]. Moreover, the virus can be transferred from mother to fetus across the placenta [4].

Mpox is generally a self-limited disease [5]. Its clinical presentation is comparable to but less severe than smallpox [5]. Symptoms include fever, headache, muscle and back pain, fatigue, and skin rash, which can be very itchy and painful [3]. Additionally, lymphadenopathy, i.e., swollen lymph nodes, was identified as a clinical feature in mpox but not in smallpox [6]. Skin lesions caused by mpox virus spread in extremities in mild cases, but in severe cases, they can spread throughout the body, including the mouth, cornea, and genitals [7]. Lesions develop simultaneously through four phases: macules to pustules, vesicles followed by pustules, and then shed off in the form of crust [8]. After an incubation period of 5 to 21 days, it might take up to 4 weeks for symptoms to subside [9].

Several observational studies have found that antibodies generated from the previous smallpox vaccine can provide up to 85% cross-protection against mpox [1,6,10]. Therefore, unvaccinated individuals have a higher incidence of mpox viral infection [11].

The WHO reported an outbreak in 2022 [6], as hundreds of mpox cases were reported outside Western and Central Africa, which has raised concerns among public health authorities worldwide [6]. On 15 May 2022, four additional cases were identified in the United Kingdom with a vesicular rash among men [4]. Crucial aspects of mpox prevention involve isolating the infected patient, practicing hand sanitizer, vaccination, and using a face mask when coming into contact with an infected animal or person [1].

Improving public knowledge regarding human mpox, signs and symptoms, prevention, treatment, and transmission should be implemented to limit the spread of this emerging disorder [3]. The Ministry of Health in Jordan announced the registration of the first case of mpox on 8 September 2022 of a thirty-year-old man who traveled to several European countries and returned to Jordan. Two previous studies were conducted in Jordan to assess Jordanian students’ and healthcare workers’ knowledge about human mpox and evaluate their conspiratorial attitude towards this emerging viral infection. Both studies showed that participants had insufficient knowledge about the disease and that conspiracy beliefs were prevalent [3,12]. Hence, in this study, we sought to assess public knowledge and perceptions of human mpox in Jordan.

## 2. Materials and Methods

### 2.1. Study Design and Study Participants

This study followed a descriptive cross-sectional design conducted using an online survey. The study followed the Strengthening the Reporting of Observational Studies in Epidemiology (STROBE) statement for cross-sectional studies (Supplementary Materials) [13]. The study aimed to assess public knowledge and perceptions of human mpox in Jordan. Participants’ recruitment and was conducted using a convenience sampling technique. All participants were approached through social media portals (Facebook and WhatsApp groups). Any participant aged 18 years or older, residing in Jordan, and with access to social media platforms considered eligible to participate in this study.

The sample size was estimated using Raosoft ^®^ sample size calculator for online survey using the following formula: n = P × (1−P) × z^2^/d^2^. With a margin of error (d) of 5%, confidence level of 95%, and a response distribution (P) of 50%, the minimum recommended sample size was 385 participants.

### 2.2. Questionnaire Development and Validation

The online survey was developed after reviewing related validated surveys in the literature [3,4,12,14]. A draft questionnaire was designed, then examined for fitness of purpose and face validity by a group of experts in observational studies. Following this review, the final version of the questionnaire was translated into Arabic. The translation was performed using the translation and back translation approach. Then, the questionnaire was piloted in a sample of 10 volunteers to verify its comprehension, clarity, and cultural acceptability before moving on to the primary survey. The data obtained from the pilot test were not included in the final data analysis. The survey contained multiple-choice questions and was designed to be completed within 10 min. The final version of the questionnaire survey was composed of four main sections. The first section included socio-demographic questions about participants. The second section assessed participants’ fears of human mpox infection. The third section evaluated participants’ knowledge about human mpox and evaluated their source of knowledge about the disease, while the last section assessed their perception towards human mpox. A five-point Likert scale (5: strongly agree, 4: agree, 3: neutral, 2: disagree, and 1: strongly disagree) was used to record the participants’ perceptions in the last section. The internal consistency and reliability of the perception section were assessed using Cronbach’s α, with a value of 0.881, which indicates excellent internal consistency.

For the knowledge section, participants gained one point for each correct statement identified and zero points for each incorrect answer. Then, the total knowledge score was calculated out of 15.

### 2.3. Data Collection

The study was conducted in Jordan between September 2022 to October 2022. The online survey was uploaded on Google Forms platform. Then, it was distributed to different Facebook and WhatsApp groups related to researchers’ networks. A written participant consent statement “Your participation in completing this questionnaire is highly appreciated” was given to the participants at the beginning of the study. If the participants were willing to proceed with the survey, they approved their consent. If not, they selected “disagree to participate” and did not continue with the survey questions. Potential participants who completed the survey were considered to have given informed consent for their participation in the study. The participants’ names were not requested, so the anonymity of respondents was preserved. To maintain confidentiality, the entire data file was downloaded and saved on the investigator’s computer.

### 2.4. Ethical Consideration

The ethics committee at Applied Science Private University approved the protocol of the current study (Approval number 2022-PHA-34). The Declaration of Helsinki guideline, along with all its amendments and revisions, was followed [15].

### 2.5. Data Analysis

The completed surveys were extracted from Google Forms as an Excel sheet. They were then exported to Statistical Package for Social Sciences version 22.0 (IBM Corp. Released 2013. IBM SPSS Statistics for Windows, Version 22.0. Armonk, NY, USA: IBM Corp) for the statistical analysis. The frequency and percentages were used for categorical variables, while the means and standard deviations were used for continuous variables.

To assess predicators affecting participants’ knowledge about human mpox (knowledge score as the dependent variable), univariate and multivariable linear regressions were used. Variables that were found to be significant on a single predictor level (*p* ≤ 0.25) using univariate linear regression analysis were entered into multivariate linear regression analyses. Variables independence was checked using person correlation where r < 0.9 indicates the absence of multicollinearity between the independent variables in regression analysis. Statistical significance was considered at *p* ≤ 0.05.

## 3. Results

In this study, 1342 participants were approached through the online distribution of the survey; among them, 20 participants were below 18 years old and were excluded, while 127 disagreed with providing the consent form and did not fill-out the survey. Thus, we ended up with 1195 participants who agreed to participate in this study and filled out the study survey. The participants had a median age of 33.0 years (IQR = 20.0), and more than half were females (n = 671, 56.2%). More than half of the participants (n = 645, 53.9%) had a graduate or postgraduate degree, and 60.3% (n = 720) were married. Furthermore, more than half of the participants (n = 704, 58.9%) reported having a monthly income of less than 400 JD (564 US dollars), and 56.0% of them (n = 669) reported having children. Nearly two-thirds of the participants (n = 625, 60.7%) were from the north of Jordan, with only 14.9% having (n = 178) reported having chronic diseases. For more details regarding the sociodemographic characteristics of the study participants, refer to Table 1.

Participants were asked about their awareness and fear of human mpox (Table 2), and only 18.7% of them (n = 224) recognized that human mpox cases were recorded in Jordan. Besides, 44.8% of the participating individuals (n = 535) believed that the transmission of human mpox is a conspiracy. In addition, less than half of the participants (n = 518, 43.3%) reported being afraid of this disease, 36.2% of them (n = 432) reported being afraid to visit any family members or friends due to human mpox. Moreover, 40.7% of the participants (n = 486) were afraid to travel to any country due to human mpox disease, and around half (n = 606, 50.7%) recommended that health officials start the vaccination campaign against human mpox infection.

Participants’ knowledge about human mpox was assessed and reported in Table 3. Participants showed a low median knowledge score (4.0 out of 15.0, IQR = 5.0). Only 27.9% of the participants (n = 334) correctly identified that human mpox is not a bacterial disease, and around half of them (n = 589, 49.3%) knew that this disease affects both genders, not only males. Considering the mode of transmission, 62.2% of the participants (743) correctly knew that human disease pops can be transmitted between individuals, and only 29.3% of them (n = 350) knew that it could be transmitted through a bite of an infected monkey. Regrinding the symptoms of the disease, only 20.4% of the participants (n = 244) recognized that mpox and smallpox have the same signs and symptoms, and around one-quarter of them (n = 305, 25.5%) knew that flu-like syndrome is one of the early signs or symptoms of human mpox. Moreover, 45.0% (n = 538), 27.6% (n = 330), and 20.2% (n = 241) of the participants identified that skin rash, vesicles, and lymphadenopathy were among the sign and symptoms of human mpox, respectively. In comparison, only 11.3% of them (n = 135) correctly knew that diarrhea is not among the symptoms of the disease. Regarding the management and prevention of this disease, only 12.0% of the participating individuals (n = 143) recognized that this disease is not treated with antibiotics. In addition, 41.3% of the participants (n = 493) knew that hand sanitizers and face masks are essential in preventing human mpox, and around one-quarter of them (n = 301, 25.2%) recognized that the chickenpox vaccine they received in their childhood does not protect them from human mpox.

**Figure 1:**
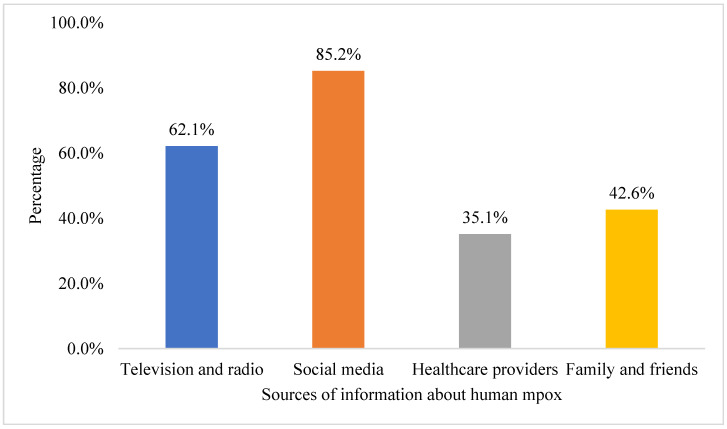
Sources of participants’ information about human mpox (n = 1195).

Regarding participants’ sources of information about human mpox (Figure 1), social media was the most-used source of information (n = 1018, 85.2%), followed by television and radio (n = 742, 62.1%), and family and friends (n = 509, 42.6%). At the same time, healthcare providers were the least used source of information by the study participants (n = 420, 35.1%).

Participants were asked to express their perceptions towards human mpox (Table 4). Results showed that more than 69% of the study participants (n = 824) believed that the world’s population could control mpox worldwide. Also, 65.0% (n = 777) agreed that the Jordanian Ministry of Health and the local population could control the mpox locally. Additionally, 57.3% of the participants (n = 685) believed that mpox would affect social and economic life like the COVID-19 pandemic, and 67.5% of them (n = 807) thought that it could add a new burden on the healthcare system of the affected countries. Besides, 71.4% of the participants (n = 853) believed that reporting symptoms of mpox to local health authorities is essential to prevent further disease transmission, and 61.8% of them (n = 739) thought that the mpox vaccine should be offered at no cost. For more details, refer to Table 4.

Lastly, univariate and multivariate linear regression analyses were performed to evaluate factors associated with participant knowledge about human mpox (Table 5). Results showed that those participants with a higher monthly income (≥400 JD/month) have higher knowledge about human mpox compared to those with lower income (<400 JD/month) (Beta = 0.062, *p* = 0.047).

## 4. Discussion

Since the start of the mpox outbreak, the number of reported cases exceeded 77,000 laboratory-confirmed cases worldwide [16]. However, the cases reported in the Middle East were limited. Due to that, only 18.7% of the participants in this study recognized that a case of mpox was reported in Jordan for a citizen who was coming from Europe [17]. Additionally, 44.8% of the participants believed that the transmission of human mpox is a conspiracy, which can explain their lack of interest in following up on news and information regarding mpox. A recent study in Jordan revealed a high prevalence of belief in conspiracies surrounding emerging viral infections and the accompanying intervention measures among the general population, which is consistent with the results of this study [3].

On the other hand, around 50% of the participants feared mpox and were afraid of visiting other people or traveling to other countries to avoid catching the infection. It was noted in previous studies that epidemics and pandemics cause fear in the general population and restrict their activities [18,19,20]. Fear and anxiety from mpox spread were reported among the Iraqi population in Kurdistan [21] and Saudi Arabia [22]. But, in Saudi Arabia, mpox caused less worry than COVID-19 among the general population [22].

In the current study, the participants showed a low median knowledge score (4.0 out of 15.0, with around half of them knowing the mpox mode of transmission and its symptoms. The general population’s knowledge about mpox was assessed in various studies, including in the Kurdistan-region of Iraq, Lebanon, and Czech, where it was found that those studies participants had insufficient knowledge [21,23,24]. While in studies conducted in Saudi Arabia and Jordan, the participants had satisfactory knowledge [3,4,22]. In addition, a study conducted in eight Middle Eastern countries, including Egypt, Palestine, Qatar, Sudan, Oman, Syria, Yemen, and Saudi Arabia, revealed that most participants knew the causative organism of mpox and the disease protective measures. Still, most were unaware of its mode of transmission, symptoms, complications, and vaccination [20]. This study also revealed that the knowledge score positively correlated with the monthly income. This may be related to having more opportunities for education and exposure to learning environments, which end up with a higher level of awareness [25,26].

Social media was the most comprehensive source of information about mpox, as reported by the study participants. This is consistent with similar studies conducted in eight Middle Eastern countries [20], as well as in the Kurdistan region of Iraq [21], Lebanon [23] and Saudi Arabia [4]. Additionally, in Czeck, social media was the second-most utilized source of information about human mpox [24]. The increase in the reliance on social media platforms as a source of health-related information needs to be addressed by governments and healthcare providers because these platforms are not a reliable source of health information [27].

Furthermore, most study participants were confident that the world’s population and the Jordanian health authorities could control Mpox, even though it will certainly be a health and economic burden. Such perception may result from experience with COVID-19; hence, the WHO and health authorities have been empowered extensively in the last three years [28,29]. These authorities have developed surveillance profiles specialized for mpox with periodical updates to enhance awareness about mpox [29]. Our study participants believed that using preventive measures is crucial to managing this outbreak, including reporting the symptoms to the health authorities and using the vaccine. Similar aspects were also discussed by Jamil et al., as they highlighted the importance of having adequate diagnostic facilities, reactivation of routine use of Vaccinia vaccination, monitoring the transmission rate via traveling, and introducing proper surveillance efforts to mitigate the spread of mpox [30]. Moreover, enhancing the knowledge about mpox will pose a primary preventive tool against disease dissemination [31,32].

The current study should be regarded in light of several limitations, including selection and sample biases based on the sampling approach and measurement bias based on the requirement to adjust the questions utilized in the Arabic language to be culturally fit. The majority of the participants were living in the north of Jordan, which might not be representative of the entire population residing in Jordan.

## 5. Conclusions

We found that the general population in Jordan had limited knowledge of human mpox infection, particularly in terms of its epidemiology, symptoms, and treatment. However, better knowledge was associated with having a high income. This suggests that those with access to reliable information are highly knowledgeable. Furthermore, endorsing conspiracy ideas about viral emergence could be potentially harmful, but providing adequate knowledge can mitigate this risk. This is crucial for social media platforms, often used to report emerging viral infections. Therefore, educational campaigns about human mpox should be promoted to fill the gaps in knowledge regarding this viral infection, which will be critical in improving the capacity to respond to the disease.

## Figures and Tables

**Table 1:** Sociodemographic characteristics of the study participants (n = 1195).

Parameters	Median (IQR)	n (%)
Age (years)	33.0 (20.0)	
Gender		
Female		671 (56.2)
Male		524 (43.8)
Educational level		
Not educated		22 (1.8)
School level		234 (19.6)
University students		294 (24.6)
University graduate		512 (42.8)
Post-graduate		133 (11.1)
Marital status		
Married		720 (60.3)
Others (Single, divorced, or widowed)		475 (39.7)
Monthly income *		
<400 JD		704 (58.9)
401–800 JD/month		366 (30.6)
801–1200 JD/month		73 (6.1)
>1200 JD/month		52 (4.4)
Place of residence		
Center of Jordan		346 (29.0)
North of Jordan		725 (60.7)
South of Jordan		124 (10.4)
Do you have children?		
No		526 (44.0)
Yes		669 (56.0)
Do you suffer from chronic diseases?		
No		1017 (85.1)
Yes		178 (14.9)

* 1 JD equals to 1.41 US Dollar, IQR: Interquartile range.

**Table 2:** Public awareness and level of fear of human mpox transmission (n = 1195).

Parameters	n (%)
Do we have human mpox cases recorded in Jordan?	
No	327 (27.4)
Yes	224 (18.7)
I don’t know	644 (53.9)
Do you think human mpox transmission is a conspiracy?	
No	660 (55.2)
Yes	535 (44.8)
Are you afraid of human mpox?	
No	677 (56.7)
Yes	518 (43.3)
Are you afraid to visit family members or friends due to human mpox?	
No	763 (63.8)
Yes	432 (36.2)
Are you afraid to travel to any country due to human mpox?	
No	709 (59.3)
Yes	486 (40.7)
Do you recommend that health officials start the vaccination campaign against human mpox infection?	
No	289 (49.3)
Yes	606 (50.7)

**Table 3:** Participants’ knowledge about human mpox (n = 1195).

Statement	Correct Answer	n (%)
Mpox is a bacterial disease infection	False	334 (27.9)
Mpox only affects males	False	589 (49.3)
An individual with mpox can transmit the disease to other individuals	True	743 (62.2)
Mpox could be transmitted through a bite of an infected monkey	True	350 (29.3)
Mpox is spread through bodily fluids	True	262 (21.9)
Mpox is usually a self-limited disease with symptoms lasting from 2 to 4 weeks	True	291 (24.4)
Mpox and smallpox have the same signs and symptoms	True	244 (20.4)
The flu-like syndrome is one of the early signs or symptoms of human mpox	True	305 (25.5)
Skin rash is a symptom of mpox	True	538 (45.0)
Vesicles on the skin are one of the signs or symptoms of human mpox	True	330 (27.6)
Lymphadenopathy (swollen lymph nodes) is one clinical sign or symptom that could be used to differentiate between mpox and smallpox cases	True	241 (20.2)
Diarrhea is one of the signs or symptoms of human mpox	False	135 (11.3)
Antibiotics are required in the management of human mpox patients	False	143 (12.0)
Hand sanitizers and face masks are essential in preventing mpox	True	493 (41.3)
The chickenpox vaccine I received in childhood protects me from mpox	False	301 (25.2)
Knowledge score out of 15 [median (IQR)]		4.0 (5.0)

IQR: Interquartile range.

**Table 4:** Perception about human mpox (n = 1195).

Statement	Agreed	Neutral	Disagree
	n (%)		
I am confident that the world’s population can control mpox worldwide.	824 (69.0)	268 (22.4)	103 (8.6)
I am confident that the Jordanian Ministry of Health and the local population can control the mpox locally.	777 (65.0)	274 (22.9)	144 (12.1)
I think mpox can add a new burden on the healthcare system of the affected countries.	807 (67.5)	264 (22.1)	124 (10.4)
I think mpox will affect social and economic life like the COVID-19 pandemic.	685 (57.3)	307 (25.7)	203 (17.0)
I have bad feelings toward the mpox virus that it might become a worldwide pandemic.	575 (48.1)	382 (32.0)	238 (19.9)
I think that it is dangerous to travel to the country’s epidemic with mpox	676 (56.6)	313 (26.2)	206 (17.2)
I think reporting symptoms of mpox to local health authorities is essential to prevent further disease transmission.	853 (71.4)	237 (19.8)	105 (8.8)
I think the mpox vaccine should be offered at no cost.	739 (61.8)	284 (23.8)	172 (14.4)

**Table 5:** Assessment of factors associated with participants’ knowledge about human mpox.

Parameter	Knowledge Score			
	Beta	*p*-Value ^#^	Beta	*p*-Value ^$^
Age (years)	−0.034	0.241 ^^^	−0.032	0.416
Gender Female Male	Reference 0.026	0.375	---	---
Educational level	Reference 0.031	0.385	---	---
School level or lower				
University students or higher				
Marital status	Reference 0.039	0.180^^^	0.035	0.359
Married				
Others (Single, divorced, or widowed)				
Monthly income	Reference 0.040	0.168^^^	0.062	0.047 *
<400 JD/month				
≥ 400 JD/month				
Place of residence	Reference −0.012 0.011	0.676 0.712	--- ---	--- ---
Center of Jordan				
North of Jordan				
South of Jordan				
Do you have children?	Reference −0.025	0.393	---	---
No				
Yes				
Do you suffer from chronic diseases?	Reference 0.000	0.978	---	---
No				
Yes				
Do you think human mpox transmission is a conspiracy?	Reference 0.004	0.882	---	---
No				
Yes				

1 JD = 1.41 US dollars. ^ Eligible for entry in multivariate linear regression, # Using univariate linear regression, $ Using multiple linear regression, * Significant at 0.05 significance level.

## Data Availability

The data presented in this study is available in the article.

## Supplementary Materials

The following supporting information can be downloaded at: https://www.mdpi.com/article/10.3390/tropicalmed8010041/s1, Checklist S1: STROBE checklist.
