# Supplementary material for: Public Knowledge and Perceptions about the Emerging Human Mpox in Jordan: A Cross-Sectional Study
Source: Trop Med Infect Dis. 2023 Jan 5;8(1):41. doi: 10.3390/tropicalmed8010041 (PMC9861141; doi:10.3390/tropicalmed8010041)
Supplement: Supplementary file 1 [file tropicalmed-08-00041-s001.zip › tropicalmed-2140736-supplementary.pdf]

STROBE Statement—checklist of items that should be included in reports of observational studies

	Item No.	Recommendation	Page No.	Relevant text from manuscript
**Title and abstract**	1	(*a*) Indicate the study's design with a commonly used term in the title or the abstract	Page 1	Lines 2-3
		(*b*) Provide in the abstract an informative and balanced summary of what was done and what was found	---	---
**Introduction**				
Background/rationale	2	Explain the scientific background and rationale for the investigation being reported	Pages 1-2	---
Objectives	3	State specific objectives, including any prespecified hypotheses	Page 2	Lines 74-75
**Methods**				
Study design	4	Present key elements of study design early in the paper	Page 2	Lines 78-90
Setting	5	Describe the setting, locations, and relevant dates, including periods of recruitment, exposure, follow-up, and data collection	Page 2	Lines 78-90
Participants	6	*Cross-sectional study*—Give the eligibility criteria, and the sources and methods of selection of participants	Page 2	Lines 85-86
Variables	7	Clearly define all outcomes, exposures, predictors, potential confounders, and effect modifiers. Give diagnostic criteria, if applicable	Page 3	Lines 111-113
Data sources/ measurement	8*	For each variable of interest, give sources of data and details of methods of assessment (measurement). Describe comparability of assessment methods if there is more than one group	Page 3	Lines 114-125
Bias	9	Describe any efforts to address potential sources of bias	Page 10	Lines 291-295
Study size	10	Explain how the study size was arrived at	Page 2	Lines 87-90

Continued on next page

Quantitative variables	11	Explain how quantitative variables were handled in the analyses. If applicable, describe which groupings were chosen and why	Page 3	Lines 130-142
Statistical methods	12	(*a*) Describe all statistical methods, including those used to control for confounding	Page 3	Lines 130-142
		(*b*) Describe any methods used to examine subgroups and interactions	NA	NA
		(*c*) Explain how missing data were addressed	NA	NA
		(*d*) If applicable, describe analytical methods taking account of sampling strategy	NA	NA
		(*e*) Describe any sensitivity analyses	NA	NA
**Results**				
Participants	13*	(a) Report numbers of individuals at each stage of study—eg numbers potentially eligible, examined for eligibility, confirmed eligible, included in the study, completing follow-up, and analysed	Page 4	Lines 144-148
		(b) Give reasons for non-participation at each stage	Page 4	Lines 144-148
		(c) Consider use of a flow diagram	---	---
Descriptive data	14*	(a) Give characteristics of study participants (eg demographic, clinical, social) and information on exposures and potential confounders	Page 4	148-155
		(b) Indicate number of participants with missing data for each variable of interest	NA	NA
		Cross-sectional study—Report numbers of outcome events or summary measures		
Main results	16	(*a*) Give unadjusted estimates and, if applicable, confounder-adjusted estimates and their precision (eg, 95% confidence interval). Make clear which confounders were adjusted for and why they were included	NA	NA
		(*b*) Report category boundaries when continuous variables were categorized	NA	NA
		(*c*) If relevant, consider translating estimates of relative risk into absolute risk for a meaningful time period	NA	NA

Continued on next page

Other analyses	17	Report other analyses done—eg analyses of subgroups and interactions, and sensitivity analyses	NA	NA
**Discussion**				
Key results	18	Summarise key results with reference to study objectives	Page 9	Lines 237-241
Limitations	19	Discuss limitations of the study, taking into account sources of potential bias or imprecision. Discuss both direction and magnitude of any potential bias	Page 10	Lines 291-295
Interpretation	20	Give a cautious overall interpretation of results considering objectives, limitations, multiplicity of analyses, results from similar studies, and other relevant evidence	Pages 9-11	---
Generalisability	21	Discuss the generalisability (external validity) of the study results	Page 10	Lines 291-295
**Other information**				
Funding	22	Give the source of funding and the role of the funders for the present study and, if applicable, for the original study on which the present article is based	NA	NA

*Give information separately for cases and controls in case-control studies and, if applicable, for exposed and unexposed groups in cohort and cross-sectional studies.

Note: An Explanation and Elaboration article discusses each checklist item and gives methodological background and published examples of transparent reporting. The STROBE checklist is best used in conjunction with this article (freely available on the Web sites of PLoS Medicine at http://www.plosmedicine.org/, Annals of Internal Medicine at http://www.annals.org/, and Epidemiology at http://www.epidem.com/). Information on the STROBE Initiative is available at www.strobe-statement.org.
